# The surface reactivity of iron oxide nanoparticles as a potential hazard for aquatic environments: A study on *Daphnia magna* adults and embryos

**DOI:** 10.1038/s41598-018-31483-6

**Published:** 2018-08-29

**Authors:** Massimiliano Magro, Marco De Liguoro, Eleonora Franzago, Davide Baratella, Fabio Vianello

**Affiliations:** 1 Department of Comparative Biomedicine and Food Science, University of Padua, Agripolis, Viale dell’Università 16, 35020 Legnaro, Italy; 2 Regional Centre of Advanced Technologies and Materials, Department of Physical Chemistry and Experimental Physics, Faculty of Science, Palacky University, 17 Listopadu 1192/12, 771 46 Olomouc, Czech Republic

## Abstract

Nano-ecotoxicology is extensively debated and nanomaterial surface reactivity is an emerging topic. Iron oxide nanoparticles are widely applied, with organic or inorganic coatings for stabilizing their suspensions. Surface active maghemite nanoparticles (SAMNs) are the unique example of naked iron oxide displaying high colloidal and structural stability in water and chemical reactivity. The colloidal behavior of SAMNs was studied as a function of the medium salinity and protocols of acute and chronic toxicity on *Daphnia magna* were consequently adapted. SAMN distribution into the crustacean, intake/depletion rates and swimming performances were evaluated. No sign of toxicity was detected in two model organisms from the first trophic level (*P*. *subcapitata* and *L*. *minor*). In *D*. *magna*, acute EC_50_ values of SAMN was assessed, while no sub-lethal effects were observed and the accumulation of SAMNs in the gut appeared as the sole cause of mortality. Fast depuration and absence of delayed effects indicated no retention of SAMNs within the organism. In spite of negligible toxicity on *D*. *magna* adults, SAMN surface reactivity was responsible of membrane bursting and lethality on embryos. The present study offers a contribution to the nascent knowledge concerning the impact of nanoparticle surface reactivity on biological interfaces.

## Introduction

As nanotechnology starts to move into large scale production, inevitably nanoscale products and by-products will enter into the aquatic environment. This makes imperative the development of reliable risk assessment procedures to deal effectively with potential ecotoxicological hazards. However, our knowledge of the harmful effects of nanomaterials is limited, particularly on aquatic animals^1^. Due to controversial results and data that remain limited, the available literature does not yet constitute a solid scientific background for approaching this emerging potential hazard, both in terms of investigative methodologies and overall knowledge.

Some studies have evaluated the consequences of the exposure of algae, mussels, amphibians, zooplankton (*Daphnia spp*.) and fish to various nanomaterials, such as silver, silica and palladium nanoparticles^2–6^. Studies on fishes reported on potentially lethal effects depending on the type of nanomaterial^7^. Sub-lethal effects on aquatic organisms have also been reported^8,9^, evidencing the occurrence of oxidative stress, changes of trace element composition in tissues, respiratory alterations, as well as modifications involving gill, liver, and gut function and morphology^10–12^. Moreover, investigations on zebrafish (*Danio rerio*) found that embryos are sensitive to metal and metal oxide nanomaterials^13,14^. Nevertheless, an adequate explanation of these phenomena is still lacking.

The food network is recognized as the main route of nanomaterial exposure for aquatic organisms^15,16^, and aggregation and deposition phenomena have a profound effect on the transport and availability of nanoparticles in the aqueous environment. In fact, it is not possible to generalize the behavior of these materials due to the wide differences of their colloidal stability and surface chemistry^17^, which depend on pH, salinity (ionic strength) and hardness of the medium. Furthermore, among the nanoparticle properties, it is hard to find out the specific feature responsible of the nanomaterial ecotoxicity. As nanoparticles are, at the same time, physical objects, possible sources of inorganic ions (due to degradation phenomena), and chemicals (due to their surface chemistry), the attribution of the main origin of hazard is tricky^18,19^. First of all, ecotoxicity effects may be ruled by the physical form of nanomaterials and/or the presence of dissolved species^20,21^, which may contribute to toxicity through different modes of action. This can introduce confounding factors for the assessment of the eco-toxicology of nanomaterials, which possibly shadow the specific effects of nanoparticles. For instance, the release of dissolved metal ions or molecules from the nanoparticle surface to the media can be the responsible of nano-toxicity^20,21^. Indeed, it is recommended that already-established models for the speciation of trace metals should be used to account for the effects of the dissolved metal fraction^22–25^, thus facilitating the interpretation of a possible nanomaterial effect. On these bases, novel findings should be evaluated as to whether nanoparticles can physically interact with test organisms, undergo dissolution in the aqueous media, and internalize/discretely localize in/on the test organisms. It is noteworthy that particle numbers or specific factors related to surface area have been suggested as more appropriate dose metrics^26^ by several studies^27–30^, envisaging the emerging relevance of available nanoparticle surface. Therefore, although nanoparticle toxicity can be attributed to relatively nonspecific biological responses to material size, shape, and bio-persistence, it can be also the result of specific biological interactions generated from reactive surfaces^31^.

For most iron oxide nanoparticles, a stabilizing coating must be provided with polymeric shells (dextran, polyvinyl-alcohol, polyethylene-glycol)^32^, thin layers of inorganic metals (gold), nonmetals (carbon), or oxides (SiO_2_)^33^, or surfactants^34^. Alternatively, the surface of nanoparticles may be modified by the use of organic molecules bearing iron chelating functionalities, such as phosphate, catechol or carboxylate groups^35,36^. Indeed, as the preparation of stable colloidal suspensions remains a significant challenge for preparative nanotechnology^37^, the employment of stabilizing coatings involves the screening of nanoparticle surface by substitution with the properties of the coating shell. On the other hand, the evaluation of the toxicity of unmodified iron oxide nanoparticles in the aquatic environment could generate a paradox as their poor colloidal stability can compromise the reliability of common tests, which assume the solubility as an implicit requisite^38^.

Surface Active Maghemite Nanoparticles (SAMNs) stand out among iron oxide nanomaterials for their exceptional colloidal stability in the absence of any coating or surface modifier, leading to excellent cellular uptake^39^, low toxicity^40^, excellent contrast agent properties for magnetic resonance imaging (MRI)^41^, and their application as magnetic carriers for biomolecules^42^. These unusual characteristics can be usefully applied to deepen our knowledge on the effects of metal oxide nanomaterials on biological systems, without any interference by organic or inorganic shells.

In the present study, *Daphnia magna*, a recognized keystone species in the food networks of many continental water bodies and an important model for ecotoxicological research^43^, was chosen as the reference organism to evaluate the effects of SAMN exposure. As a corollary, two growth inhibition tests were performed respectively on the green alga *Pseudokirchneriella subcapitata* and on the vascular plant *Lemna minor*.

## Results

The main difference between nanoparticles and dissolved chemicals is that the former are solid objects with a defined physical volume and shape, and hence ruled to a great degree by physical forces^44^. It is currently assumed that the toxicity of nanomaterials is partly, and in some cases fully, attributable to dissolved metal ions and metal complexes. However, beyond defining the physical shape of nanoparticles and establishing the boundary with their surrounding liquid, surfaces represent the chemical counterpart of these objects. Actually, the surface defines the final properties of nanomaterials and is eventually responsible of the multitude of applications in several scientific and technological areas.

The structural integrity of SAMNs was the object of several studies^45^, hence, thanks to the robustness of stoichiometric pure maghemite nanocrystals, their dissolution can likely be excluded as a factor determining toxicity. At the same time, due to crystal truncation at the boundary with the solvent, SAMNs expose undercoordinated iron sites, comparable to some extent to free iron(III) in solution as recently employed for evaluating the role of the iron(III) receptor in *Pseudomonas fluorescens*^46^. Thus, SAMNs represent an ideal model as their surface can display chemical reactivity without releasing iron ions.

### Colloidal stability of SAMNs

Nanoparticle dissolution and aggregation are assumed to control the toxicity of nanoparticles^47–49^. However, the precise implications of these factors are difficult to assess^50^. The monitoring of nanoparticle behavior, along with the exposure to biological models, would ideally require a multiple point characterization, which can hardly be carried out without interfering with the living organisms^51^. Thus, the colloidal stability of SAMNs was studied under two standardized solutions suitable for *D*. *magna* culturing, namely in Rocchetta (oligomineral) water and in “Aachener Daphnien Medium” (ADaM)^52^.

The strong interaction with the solvent makes SAMNs an elective model for studying the effect of bare iron oxide nanoparticles on aquatic organisms. Available zeta-potential values (*ζ*) of nanostructured iron oxides, at neutral pH, are generally close to zero^53^, explaining the very low colloidal stability of the suspensions of these nanomaterials. Conversely, naked SAMNs present a remarkable positive *ζ* value (+38.7 ± 8.7 mV, conductivity 0.00347 mS cm^−1^)^42^, responsible for the strong electrostatic repulsion between nanoparticles and conferring a unique stability to naked SAMNs as colloidal suspensions in water. In fact, in a manner similar to hydrous ferric oxides, the coordination of water molecules on SAMN surface leads to positively charged OH_2_^+0.5^ groups^54^. As a matter of fact, a nanoparticle suspension is far from a solution of chemicals and its overall stability is ruled by the characteristics of both the particle and the medium^55,56^. This is true even in the case of SAMNs, which can be destabilized by saline solutions. In fact, the local ion concentration affects the colloidal stability of nanoparticles owing to Debye-Hückel screening^57^.

**Figure 1:**
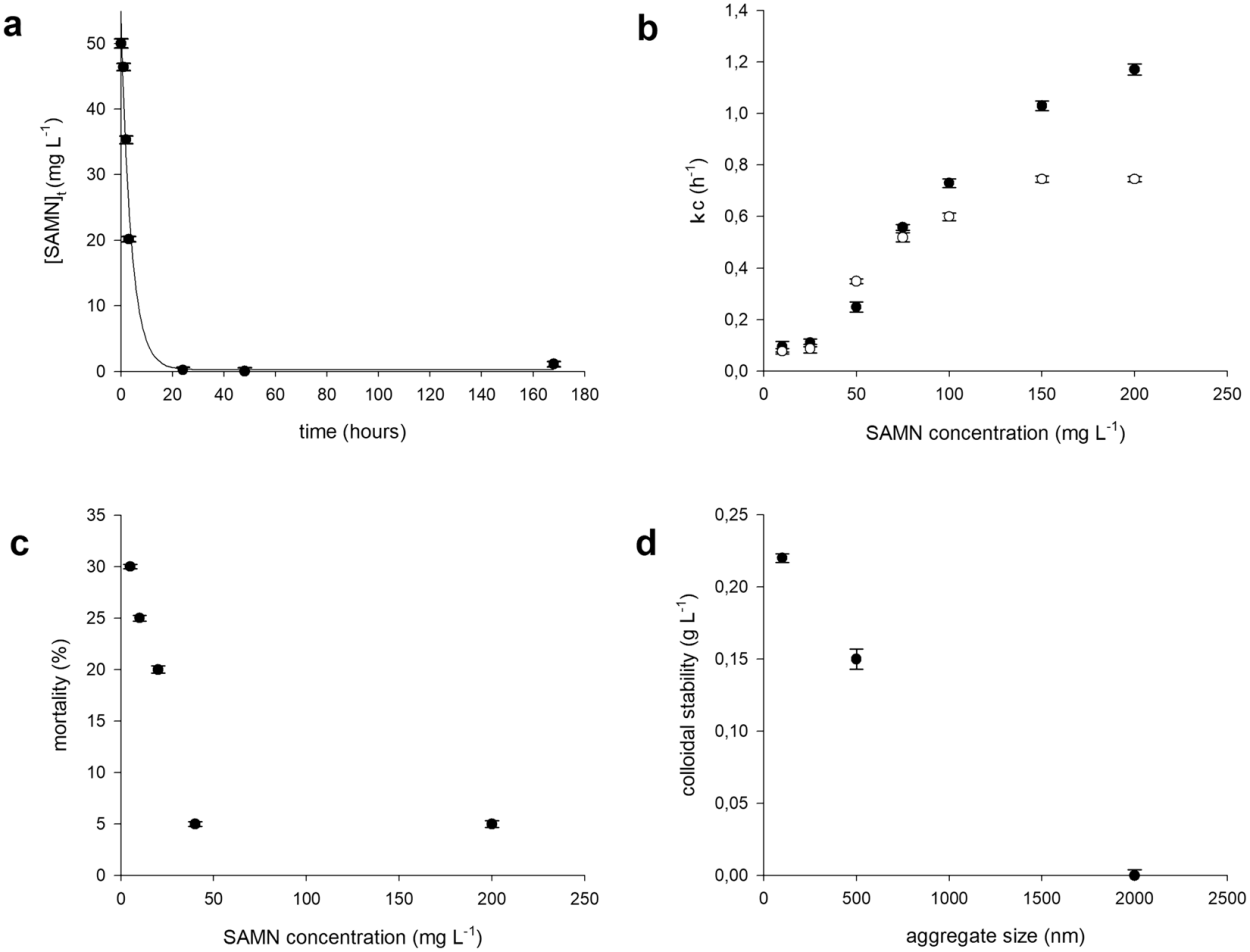
Behavior of SAMNs in water as colloidal suspension. (**a**) Coagulation kinetics of SAMNs in ADaM and Rocchetta media at 50 mg L^−1^ SAMN; (**b**) concentration dependence of SAMN coagulation kinetic constants in ADaM (○) and Rocchetta (●) medium; (**c**) *D*. *magna* mortality as a function of SAMN concentration in the absence of agitation in Rocchetta medium; (**d**) colloidal stability of SAMNs in Rocchetta medium as a function of the aggregate size determined by dynamic light scattering.

The study was carried out using the concept of the Critical Coagulation Concentration (CCC)^58^. The CCC value of nanomaterials is defined as the minimum concentration of counterions to induce the aggregation of a colloidal particle suspension^59^. In the present case, experiments were carried out at constant salinity, thus, the minimum concentration of SAMNs leading to the colloid coagulation was assumed as the CCC. The colloidal coagulation of SAMNs was studied by UV-Vis spectroscopy, monitoring the typical nanoparticle maximum absorbance at 400 nm (ε = 1520 M^−1^ cm^−1^), as a function of time (t). For all tested SAMN concentrations, in the range between 10.0 and 200.0 mg L^−1^ SAMN, the absorbance decreased following an exponential decay in both media (see Fig. 1a), and the corresponding first order kinetic constants were calculated according to the following eq. (1):

1 $${[{\rm{SAMN}}]}_{{\rm{t}}}={[{\rm{SAMN}}]}_{\infty }+{[{\rm{SAMN}}]}_{{\rm{o}}}\times {{\rm{e}}}^{-({\rm{kc}}\times {\rm{t}})}$$

where [SAMN]_t_ stands for SAMN concentration at the instant t, [SAMN]_∞_ and [SAMN]_0_ correspond to SAMN concentration at t = ∞ and at t = 0, and k_c_ is the first order coagulation kinetic constant.

In both media, the calculated coagulation kinetic constant (k_c_) depended on SAMN concentration. The values of k_c_ were plotted as a function of SAMN concentration in order to individuate the CCC in Rocchetta and ADaM media, leading to sigmoidal trends and evidencing the transition from fast to slow aggregation regime (see Fig. 1b). The inflection points resulted at 60 mg L^−1^ SAMN and 100 mg L^−1^ SAMN for Rocchetta and ADaM media, respectively. Thus, Rocchetta emerged as the preferred medium for the dispersion of SAMNs in the whole range of concentrations tested. Nevertheless, as coagulation rates increased at concentrations higher than 25.0 mg L^−1^ SAMN, this value was considered as the CCC of colloidal suspensions of SAMNs in Rocchetta medium.

### Acute toxicity test on *D. Magna*

In recent years, SAMNs were proposed for biotechnological applications and their cytotoxicity was extensively studied *in vitro* on eukaryotic cells, such as HeLa cells^40^, mesenchymal stromal cells (MSCs) from rat and human^39^, mesenchymal stem cells from horse peripheral blood^42^ and on prokaryotic cells, such as *Salmonella typhimurium*^60^ and *Pseudomonas fluorescens*^46^. This body of work laid the basis for moving to an animal model.

*Daphnia magna*, as a freshwater invertebrate, is considered one of the most sensitive organisms for toxicity evaluation^61^. Preliminary, acute toxicity tests with SAMNs on *D*. *magna* were performed in the absence of agitation in the whole range of concentrations explored in the previous paragraph, hence overcoming the CCC. We observed that the fraction of immobilized daphnids decreased as SAMN concentration approached the CCC (see Fig. 1c), and was zeroed at a SAMN concentration approximatively equal to the sigmoid inflection point of the aggregation kinetic curve (see Fig. 1b). These data evidenced the strong influence of the colloidal behavior of the nanomaterial on the animal response and the observed decrease of SAMN effects can be attributed to the progressive unavailability of SAMNs in the medium.

The guidance documents for aquatic ecotoxicity assessment of chemicals recommend the use of concentrations below the limit of their water solubility^62^. Thus, this concept must also be applied to nanoparticles. Therefore, the final acute tests were performed below the CCC value of SAMNs (25.0 mg L^−1^ SAMN), with the awareness that, according to literature, this still represents a high concentration for nanomaterials^63^. The behavior and fate of nanoparticles could be due to physical factors, such as the mere overloading of the nanomaterial in the test organisms, which could lead to effects different from the actual toxic response of a chemical compound. In crustaceans, nanomaterial overloading can alter the feeding behavior or impair animal mobility^64,65^. These physical effects, of course, need to be accounted for and possibly, eliminated. Nevertheless, despite the extensively debated relevance of the high nanomaterial concentrations into the natural environment^66^, attempts to reveal nanoparticle toxicity have been performed at concentrations up to two orders of magnitude above the CCC used in the present study^67^.

In the acute toxicity test on *D*. *magna*, no response was observed at the lowest SAMN concentration (acute NOEC = 1.25 mg L^−1^ SAMN) with respect to controls. The fitting of the experimental data did not match the ideal sigmoid dose-response curve. However, using the Trimmed Spearman-Karber Method an EC_50_ of 3.54 mg L^−1^ SAMN (2.84–4.40 mg L^−1^ 95% C.I.) was calculated under our experimental conditions.

### SAMN distribution in *D. magna* after the acute toxicity test

**Figure 2:**
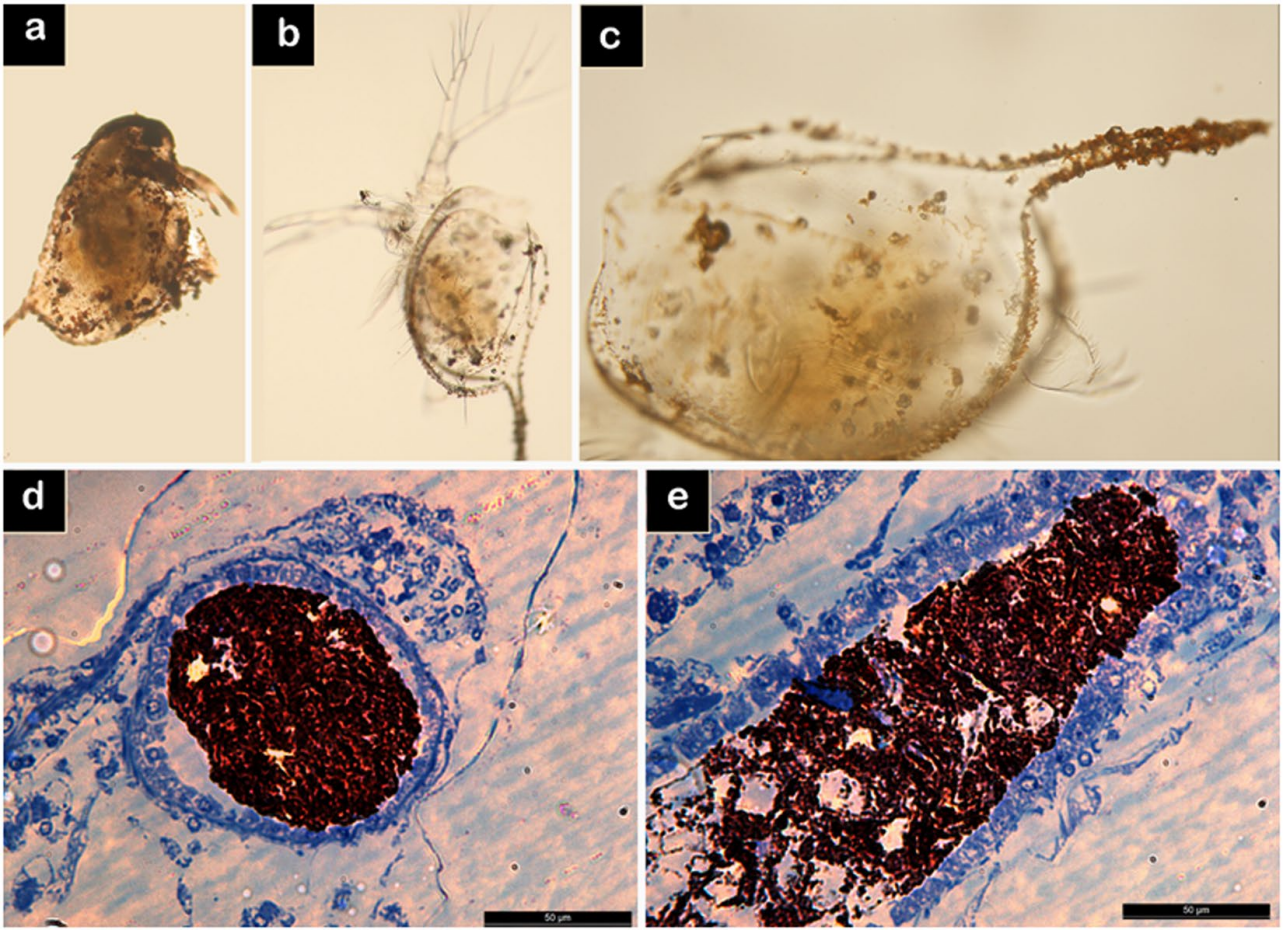
Accumulation of SAMNs in *D*. *magna*. (**a**) Aggregates of SAMNs on *D*. *magna* (20X magnification) after exposure to 10.0 mg L^−1^ SAMNs; (**b** and **c**) aggregates of SAMNs in *D*. *magna* after exposure to 1.25 mg L^−1^ SAMNs (20X and 45X magnification); (**d** and **e**) transversal and longitudinal sections of *D*. *magna* gut (50 µm size bar).

Organisms used for the acute toxicity tests were collected and observed by optical microscopy to study nanoparticle ingestion and external deposition^68–70^. In the range of SAMN concentrations tested, microscopy images showed the appearance of nanomaterial aggregates on the carapace surface. The amount of deposited material increased with SAMN concentration (Fig. 2a). At the lowest SAMN concentration (1.25 mg L^−1^ SAMN), the nanomaterial aggregated mainly on the tail spine (Fig. 2b,c). Likely, the crustacean surface acted as a nucleation seed for SAMN aggregation, and the phenomenon proceeded as a response to the destabilization of the colloid caused by the salinity of the medium.

The physical attachment of nanoparticles to the *D*. *magna* carapace has already been documented for TiO_2_^65^ and CeO_2_^71^ nanoparticles, and correlated to sub-lethal toxicity effects.

Optical microscopy of the transversal and longitudinal sections of daphnids showed the presence of large macro aggregates in the gut lumen (Fig. 2d,e), occupying the whole volume of the intestinal section. In order to express the amount of total protein over the nanoparticle mass in *D*. *magna* samples, the determination of protein content^72^ was also performed, and resulted of 7.7 mg SAMN g^−1^ protein.

**Figure 3:**
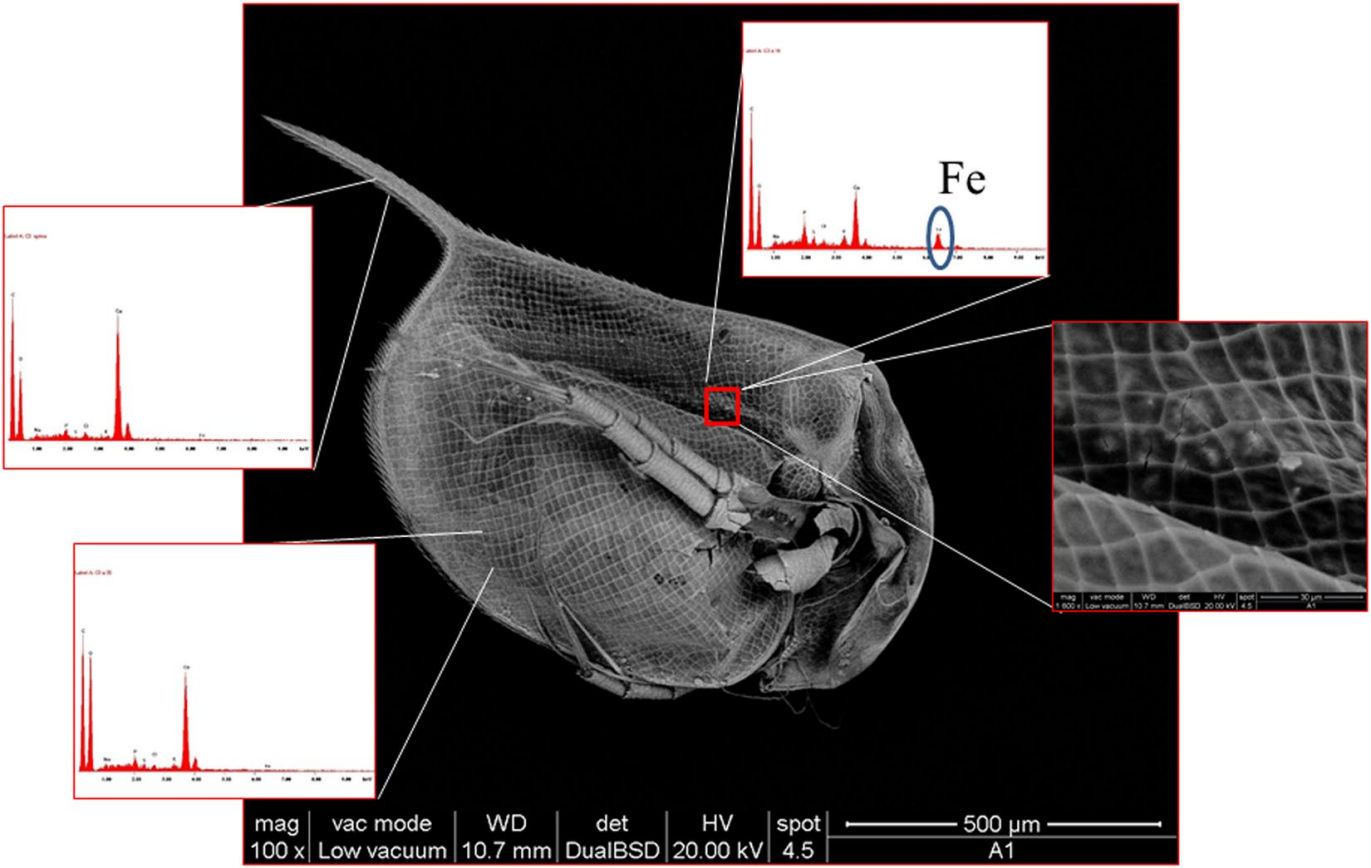
SEM micrograph of *D*. *Magna* after incubation in the presence of 1.25 mg L^−1^ SAMNs. Insets: EDX elemental analysis and higher magnification of gut detail.

Scanning electron microscopy (SEM) equipped with an EDX (Energy-dispersive X-ray spectroscopy) detector was used to deepen the analysis about SAMN distribution into the *D*. *magna* body. After incubation in the presence of 1.25 mg L^−1^ SAMNs, daphnids were collected and prepared for SEM-EDX analysis as described in Methods. EDX spectra were collected at point locations over the whole *D*. *magna* body for the determination of the chemical composition. SEM-EDX highlighted that SAMNs were not present over the organism surface. The loss of SAMNs, observed by optical microscopy, can be attributed to the sample preparation process and indicates a labile adhesion of the nanoparticles to the *D*. *magna* carapace. Elemental analysis by EDX revealed that SAMNs were concentrated in the intestinal tract of the daphnids, as witnessed by the characteristic iron peak in the corresponding EDX spectrum (see inset of Fig. 3), and confirming the finding obtained by optical microscopy on *D magna* sections.

Finally, animal mobility was evaluated and, due to the presence of SAMNs in the gut lumen, *D*. *magna* swimming was drastically altered upon exposition to an external magnetic field (see Supplementary Movie 1).

It is worth mentioning that there are no universal parameters for the evaluation of the consequences of the mere presence of nanoparticles in the gut (without any sign of internalization). Nevertheless, literature examples reported on protrusions of the gut epithelia in fish after exposure to TiO_2_ nanoparticles^73^, reduction of food intake and alteration in gut mobility^74^.

### Kinetics of loading and depletion of SAMNs in *D. magna*

*D*. *magna* uses an active particle selection for food uptake, based on the direct interception and random internalization through filtering the surrounding water by the setulae of the thoracic limbs, which act like a comb. Based on the distance between the *setulae*^75^, nanoparticles seem too small to be collected, but the ingestion of isolated nanoparticles could still be possible through water intake^68,76^. In practice, as already reported, micron-size aggregated nanomaterials can be intercepted by the *setulae*, as observed in other freshwater cladocera^77^. In Fig. 1d, SAMN aggregates with a size around 1.0 µm can be observed, which are in the range available for *D*. *magna* filtration (0.4–40 µm)^75,78^.

**Figure 4:**
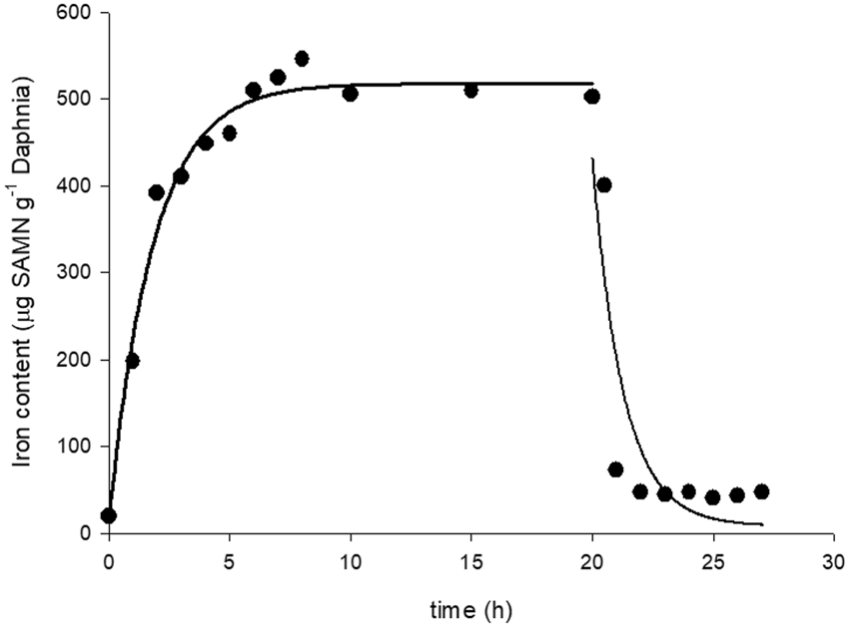
SAMN loading and depletion in *D*. *magna* as a function of time. Measurements were carried out on 170 daphnids, 14 day old, exposed to 1.25 mg mL^−1^ SAMN in Rocchetta medium. Iron content was determined by ICP-AES as described in Materials and Methods.

Inductively coupled plasma-atomic emission spectroscopy (ICP-AES) was used to monitor the loading rate of SAMNs in *D*. *magna* and, after the transfer of daphnids into pure medium, the depletion rate. In the presence of 1.25 mg L^−1^ SAMN, the rate of SAMNs uptake, expressed as µg iron per gram body mass, rapidly increased with time. In particular, the increase of iron content could be roughly approximated to an exponential growth, and the corresponding first order kinetic constant was calculated (k = 0.56 ± 0.06 h^−1^). Nanoparticle uptake reached a steady state after approximately three hours, at around 500 µg SAMN g^−1^ daphnid. Henceforward, the iron content did not vary significantly as evidenced by a final check after 24 hour exposure, suggesting the occurrence of a saturation phenomenon. Therefore, the maximum loading of SAMNs can be expressed as the mass ratio at this stationary condition (500 µg SAMN g^−1^ daphnid), which corresponds to the plateau of the uptake curve (Fig. 4). Such a significant loading is in good agreement with the available data reporting on the ingestion of other nanomaterials by the crustacean^79^, which was attributed mainly to the retention of nanoparticles in the gut lumen without absorption into the organism.

Considering that *D*. *magna*, as fine filter-feeder, can filter 18.5 mL h^−1^ ^80^, the theoretical amount of nanoparticle intake was calculated and resulted of 925 µg SAMN g^−1^ daphnid h^−1^. This value is well aligned with the observed intake rate in the first three hours incubation (see Fig. 4). Moreover, SAMN depletion rate in *D*. *magna* was studied. After the transfer of daphnids into fresh medium, iron content rapidly decreased, approximating an exponential decay, and the corresponding first order kinetic constant was calculated (k = 0.81 ± 0.30 h^−1^). After about three hours, the iron content in daphnids reached the basal level indistinguishable from controls, indicating that SAMNs were not retained by the organism. Such a complete depuration suggests the absence of significant interactions between nanoparticles and the gut epithelium. This is not trivial as SAMN retention could determine a prolonged and detrimental exposure to nanoparticles. As an example, CuO nanoparticles exerted a toxic effect on daphnids over a long post-exposure period, after animals were transferred into pure medium. This effect was attributed to the combination of the retention and progressive degradation of the nanomaterial to metal ions into the gut^81^.

Thus, a group of 20 daphnids was monitored for additional 12 days after the complete SAMN depletion in clean culture media. At a SAMN concentration just below the acute NOEC value (1.25 mg L^−1^), no delayed toxic effects were observed. The normal viability of daphnids after SAMN depletion indicates a negligible nanomaterial retention, degradation and absorption from the gut lumen and/or a good tolerance of the daphnids.

In conclusion, according to the studies on loading, distribution and depletion, SAMNs appeared mainly in the gut lumen. Nevertheless, harms consequent to the physical attachment of TiO_2_ and CeO_2_ nanoparticles have been reported in crustaceans^55,64^. Therefore, in order to deepen our knowledge on the possible adverse outcomes on daphnids correlated to the presence of SAMNs, sub-lethal effects on swimming activity, reproduction, and daily growth performances, were evaluated.

### Effects of SAMNs on swimming activity of *D*. *magna*

The swimming activity is an important behavioral feature of *D*. *magna* as it can influence ecologically relevant activities, such as predation avoidance and food supply. A decreased swimming activity was revealed for two daphnia species (*D*. *similis* and *D*. *pulex*) upon exposure to CeO_2_ nanoparticles^64^. The hopping frequency (the beating frequency of daphnia antennas) was affected upon exposure to C_60_ and a fullerene derivative^82^. As a consequence of a decreased swimming speed, the organism must expend more energy on movement, thus decreasing the energy available for growth and reproduction as observed by Gaiser *et al*.^71^. Moreover, swimming activity is closely related to the respiration rate as it generates a water current that facilitates the exchange of oxygen^83,84^.

Considering the results obtained from the loading and depletion tests, nanoparticle presence in the gut lumen represents a negligible mass increase (0.05%) for daphnids after 48 h exposure to 1 mg L^−1^ SAMN. Nevertheless, a possible interference on swimming activity, also due to the external deposition of SAMN aggregates (Fig. 2a–c), could not be excluded.

Six day old daphnids were selected as they were sufficiently developed to be filmed by a conventional video camera, but not yet ready for reproduction. Indeed, brood delivery in *D*. *magna* causes a physiological pause in swimming activity that would unavoidably alter the test results. Differently from the conditions used in the chronic tests, in which the medium was shaken for many days, shaking the medium for 48 h did not hasten the first brood delivery. Hence, at the end of the exposure to SAMNs, the 8-day old daphnids were filmed without experiencing any change of motility due to first brood delivery. No significant effects of SAMNs on the swimming activity of *D*. *magna* were observed. Average travelled distances (in three minutes) were 95.4 ± 28.1 cm (controls), 97.3 ± 45.0 cm (0.625 mg L^−1^ SAMN) and 111.3 ± 48.9 cm (1.25 mg L^−1^ SAMN). Examples of travelled paths of *D*. *magna* (control and exposed to SAMNs) are reported in Fig. S1(a,b) in Supplementary Information, and in Fig. S2 (Supplementary Information) where the amplitudes of the swimming speed of 20 individuals are presented. Concluding, after 48 h exposure to SAMN concentrations equal to or below the acute NOEC, neither SAMNs accumulation in gut lumen nor their aggregation on carapace surface influenced *D*. *magna* swimming activity, confirming the acute NOEC of 1.25 mg L^−1^ SAMN.

### Chronic toxicity of SAMNs on *D*. *magna*

Sub-lethal toxic effects at low concentrations of nanomaterials (<1.0 mg L^−1^) have already been observed in crustaceans and were related to the energy deficit associated with the depuration process of non-nutritious particles^79^.

In reproduction tests, *D*. *magna* was exposed to five scaled concentrations in the 0.078–1.25 mg L^−1^ SAMN range. Noteworthy, the effects of SAMNs on reproduction and daily growth performances were not dose dependent (see Table 1). The average growth performances of exposed groups (103 ± 6 live offspring/female and 135 ± 5 µm daily growth) were slightly lower than those of controls (127 ± 25 live offspring/female and 149 ± 23 µm daily growth), but the differences were not significant, probably for the relatively limited statistical power of the test^85^. However, the long-lasting exposure (21 days) even at concentrations equal to or below the acute NOEC led to a mortality rate of 20–60%, although without any correlation with SAMN concentration (Table 1). It should be noted that the semi-static design of the chronic test, implying the renewal of the SAMN containing medium every other day, may have led to the reiteration of SAMN loading in the gut lumen already observed in the acute toxicity tests. Hence, a progressive accumulation of iron oxide into the intestinal tract can be envisaged, probably overcoming a threshold of tolerability. Below this threshold, the presence of SAMNs did not endanger daphnids as no significant effects on reproductive activity and growth were observed, confirming the low toxicity of and/or good tolerance to SAMNs. Indeed, the reproductive activity of daphnids is usually very sensitive to any kind of disturbance as is swimming activity, which in turn can influence the food intake. None of these animal activities was affected by the presence of SAMNs in the gut lumen. It should be noted also that, curiously, a precocity of the first brood production was observed (Table 1). Since this phenomenon occurred also in the control group, it was presumably elicited by the continuous agitation of the medium, and therefore not correlated with the nanoparticles exposure.

**Table 1:** Chronic toxicity test.

Concentration (mg/L)	Mortality (%)	Precocity of first brood (%)	Total number of newborn	Live offspring/female	Daily growth (mm)
Control	20	50	1071	127	0.149
0.078	60	0	573	105	0.136
0.156	20	40	1002	108	0.128
0.313	60	30	611	96	0.133
0.625	40	10	789	98	0.140
1.25	60	40	659	108	0.139

Data on mortality, precocity of first brood production (within 7 day age), reproduction and growth of *Daphnia magna* exposed to SAMN suspensions. Mean reproductive output and daily growth per surviving parent animal are reported.

### Effects of SAMNs on *D*. *magna* embryos

It has been demonstrated that *D*. *magna* embryos in the brood chamber are directly exposed to chemicals dissolved in the aqueous environment^86^. Besides absorption, the primary source of nanoparticle hazard is assumed to be the release of dissolved metal ions. As no relevant effect of SAMNs was observed on the reproductive activity, we concluded that SAMNs are not able to enter in the brood chamber and, as expected, iron ions were not released into the surrounding environment.

**Figure 5:**
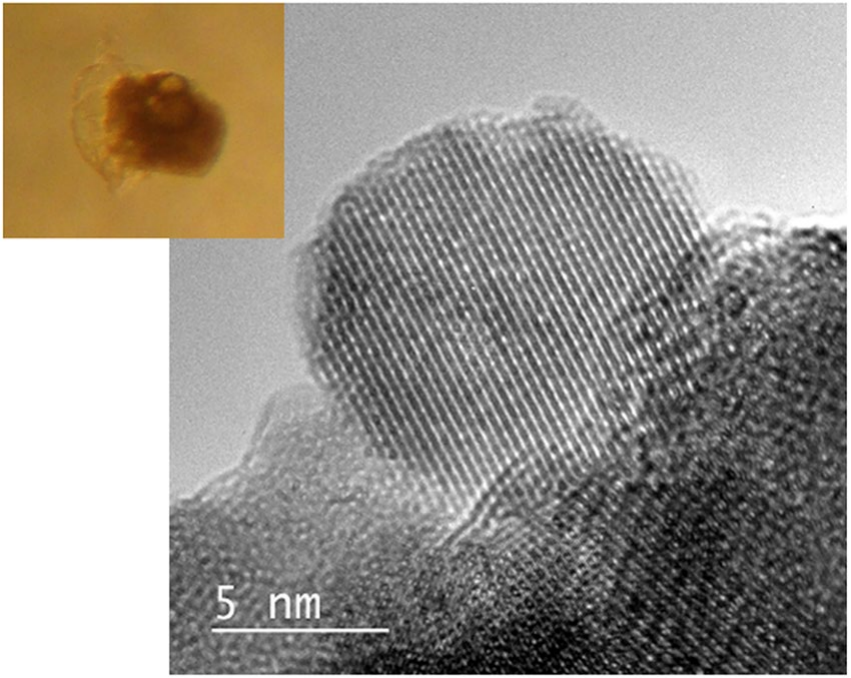
TEM micrograph of SAMNs. Inset: embryo extrusion upon incubation with SAMNs.

In order to highlight the surface reactivity of SAMNs, we used isolated embryos in an *in vitro* test as sensitive biological model. In controls, embryonic development proceeded completely within three days with 92% hatchability, and no morphological alterations were observed in individuals reaching full growth. Noteworthy, embryo mortality (48 ± 18%) was observed in the early phase of development in the presence of SAMNs, independently of concentration. In most cases, lethality was due to the extrusion of the embryo consequent to a membrane bursting (see Fig. 5, inset). Rarely, embryo growth was simply arrested with no evidence of membrane damage. It is worth noting that no sign of early embryonic maturation (head capsule, antennae, and pigmented eyes) was recognizable in the extruded embryos, indicating the precocity of the egg membrane rupture (see Fig. 5, inset). Membrane bursting cannot be attributed to medium shaking or to manipulation during the tests because the phenomenon was absent in the control group. Indeed, embryonic membranes exhibit a good resistance to mechanical injuries^87^, and the damage induced by SAMNs cannot be attributed to a mere mechanical stress. The process seems to be driven by the maximization of the contact between the biological membrane and iron oxide nanoparticles that leads to a strong adhesive interaction tearing off the cell membrane. It should be considered that when embryo development proceeded beyond the physiological shedding of the egg membrane, no adverse outcomes were observed. Thus, SAMNs exerted a detrimental effect only when the membrane was still present. Possibly, surface under-coordinated iron sites of SAMNs specifically interacted with proteins and/or phospholipids coating the outer shell of the egg membrane^88–90^. These macromolecules are both susceptible to interact with SAMNs as they present a proclivity toward Fe^3+^ chelation^40^.

Noteworthy, an analogous effect was recently reported about the toxicity of silver nanoparticles and their surface reactivity caused by crystal defects at the boundary with the solvent. Silver nanoparticles were considerably more toxic in zebrafish embryos than in adults. Note that animal injuries required the direct contact with the nanoparticles given their low tendency of releasing Ag^+^ ions into the medium^91^.

The toxicity of SAMNs on *D*. *magna* embryos could provide an important hint on the role played by the surface reactivity of nanoparticles in their interplay with biological interfaces, which actually represents the frontier in nano-toxicity studies^31^.

### Toxicity effects of SAMNs on *Pseudokirchneriella subcapitata* and *Lemna minor*

In microalgae, the relatively tough and thick cell wall acts as a barrier, and is commonly assumed to prevent nanoparticle internalization. Notwithstanding this protection, pores in the cell wall have diameters in the 5–20 nm range and their permeability is altered during cell cycling^92^.

**Figure 6:**
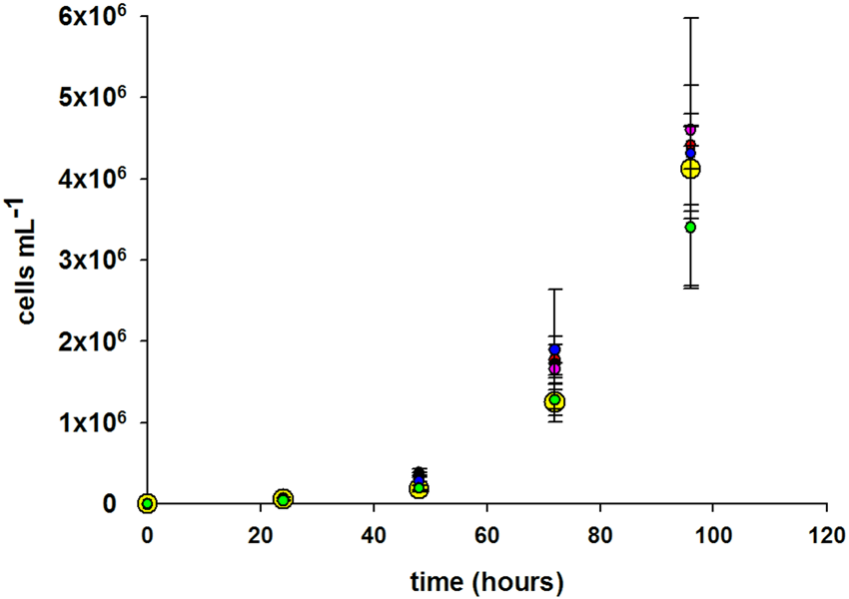
Growth curves of *P*. *subcapitata* in the presence of SAMNs. Measurements were carried out in BBM: Control (), 1.25 mg L^−1^ SAMNs (●), 2.5 mg L^−1^ SAMNs (), 5 mg L^−1^ SAMNs (), 10 mg L^−1^ SAMNs (), and 20 mg L^−1^ SAMNs ().

The toxicity of SAMNs was also tested on *P*. *subcapitata*, and the algal growth curves are reported in Fig. 6. After 48 h incubation in the presence of SAMNs, a slight and not significant inhibition of algal growth was observed both at 10 and 20 mg L^−1^ SAMNs. While this apparent effect persisted only at the highest SAMN concentration tested after 72 h incubation.

An analogous test was carried out on *L*. *minor*, and the average number of fronds, total frond area and fresh weight after one week exposure are reported in Table 2. As shown, no significant inhibition of plant growth was observed even at 20 mg L^−1^ SAMNs.

**Table 2:** *Lemna minor* growth after 7 days exposure to SAMNs.

SAMNs (mg L^−1^)	fronds	area (cm^2^)	weight (mg)
0	64 ± 6	3.61 ± 0.49	103 ± 15
1.25	63 ± 16	3.96 ± 1.25	144 ± 59
2.5	67 ± 8	4.44 ± 0.66	101 ± 59
5	67 ± 12	4.27 ± 0.95	128 ± 45
10	59 ± 9	3.73 ± 0.72	104 ± 35
20	61 ± 11	3.97 ± 1.23	127 ± 75

## Discussion

Nanotechnology has started to move to large scale production and inevitably nanoscale products will enter into the aquatic environment^93^. In fact, a rough and dated estimate reported on a world nanoparticle production of 55–55,000 ton year^−1^ for SiO_2_, 550–5,500 ton year^−1^ for TiO_2_, 55–5,000 ton year^−1^ for Al oxides, 5.5–5,500 ton year^−1^ for Fe oxides, 55–550 ton year^−1^ for ZnO, 55–550 ton year^−1^ for carbon nanotubes and 5.5–550 t year^−1^ for Ce oxides and Ag^94^. Considering the increasing magnitude of nanoparticle production, their presence in the aquatic environment is likely predicable and goes beyond the restricted number of nanoparticles above considered^95^. Nevertheless, few studies are available in literature on this topic. As an example, TiO_2_ particles (between 20 and 300 nm) are released from building facade paints by natural weather conditions and then transported by runoff into freshwaters^96^. Silver nanoparticles are massively used in commercial products, especially textiles and plastic food containers, and heavily washed away^97,98^. The large-scale application of nano-sized zero-valent iron particles (nZVI) for the remediation of contaminated soil and groundwater was proposed^99^.

Conventional bioassay methods, designed for chemicals, can hardly be applied even under modified conditions due to the differences of the interactions between molecules or ions and nanomaterials with biological systems. Moreover, the huge differences among the natures, chemical-physical properties, and coatings of the different nanomaterials require the development of new ecotoxicological paradigms^100^. Within this scenario, we focused our work on a widely used nanomaterial, namely iron oxide. Among nanostructured iron oxides without any surface modification, water dispersions of SAMNs represent the first example of stable colloids. Thus, they can be considered an elective benchmark for the evaluation of ecotoxicological effects of nanomaterials, and able to provide insights into the actual toxicity of nano-sized iron oxides.

Firstly, the effects of the salinity of the medium required for ecotoxicity tests on the colloidal stability of SAMNs, in term of critical coagulation concentration, were evaluated. Then, the effects of SAMNs on *D*. *magna*, as model aquatic organism, were assessed by optical microscopy, SEM-EDX and ICP-AES, evidencing their confinement in the gut lumen. Moreover, a kinetic study on SAMN loading and depletion was accomplished, revealing that, once moved to clean water, daphnids were able to eliminate the nanomaterial in a short time. This, together with the absence of any significant effect on the reproduction and swimming activity, suggests that no chemical interaction occurred in the adult animal. Due to the mortality observed in the reproduction tests, even at the lowest SAMN concentration (78 µg L^−1^), the definition of a chronic NOEC and the derivation of a Predicted No Effect Concentration was not possible^101^. However, it seems highly improbable that, at environmentally realistic concentrations (ng L^−1^ to µg L^−1^), SAMNs could exert any adverse effect on the crustacean population. Indeed, under natural conditions, daphnids live in lentic, oligohaline water, where a prolonged exposure to SAMNs, notwithstanding the good colloidal stability, is hard to envisage. The accumulation of SAMNs in the gut of *D*. *magna* adults appeared the sole source of hazard, and no toxicity was revealed in the other models employed: the green unicellular alga *P*. *subcapitata* and the vascular plant *L*. *minor*. In these two model organisms from the first trophic level, SAMNs did not cause any significant growth inhibition. Conversely, experiments on isolated embryos of *D*. *magna* indicated that SAMNs interact with the egg membrane causing lethality by membrane extrusion. However, this phenomenon is unlikely under natural conditions as embryos are physically protected by the brood chamber.

## Conclusions

Experimental results and theoretical interpretations reported in recent years have sparked an intense debate on the potential risks of nanotechnology. One of the hottest questions is whether nanoparticles present ecotoxicological risks for the health of the aquatic organisms.

In response to this, a growing awareness toward the complexity of nanoparticles is pushing the research to become more interdisciplinary. In this context, among the inherent properties of nanoparticles, the surface reactivity is emerging as a crucial contribution to nano-ecotoxicity. In fact, the increasing interest on nanomaterials is due to their high surface area to mass ratio. Beside this, atoms on the surface present a different reactivity with respect to those in the bulk and confer a specific chemical reactivity to the surface of the nanomaterial.

In order to shed light on the surface reactivity of nanomaterials in the view of their possible impact on biological systems, benchmarks are becoming of fundamental importance. SAMNs, as iron oxides, represent an ideal nanomaterial as their surface display a chemical reactivity without releasing metal ions into the solution.

In this work, this concept was highlighted by comparing the effects of SAMNs on *D*. *magna* adults and embryos. In *D*. *magna* adults, the toxicity of SAMNs was correlated to physical effects, which could be eliminated by a thorough washing out. Conversely, the contact of SAMNs with isolated embryos led to injuries with the consequent bursting of the external membrane.

The current study stresses the importance of the colloidal stability and the surface chemistry of nanomaterials considering the surface reactivity as an essential factor for evaluating ecotoxicological risks in the aquatic environment. It provides a contribution for laying the basis of future nano-ecotoxicological studies and stimulates the derivation of different experimental models and the modification of standard test conditions.

## Methods

### Chemicals

Chemicals were purchased at the highest commercially available purity and were used without further treatment. Iron(III) chloride hexahydrate (97%, FeCl_3_∙6H_2_O), sodium borohydride (NaBH_4_), ammonia solution (35% in water) were obtained from Aldrich (Sigma-Aldrich, Italy).

### Synthesis of iron oxide nanoparticles (SAMNs)

A typical nanoparticle synthesis has already been described^102^ and can be summarized as follows: FeCl_3_·6H_2_O (10.0 g, 37 mmol) was dissolved in MilliQ grade water (800 mL) under vigorous stirring at room temperature. NaBH_4_ solution (2 g, 53 mmol) in ammonia (3.5%, 100 mL) was then quickly added to the mixture. Soon after the reduction reaction occurrence, the temperature of the system was increased to 100 °C and kept constant for 2 hours. The material was cooled at room temperature and aged in water, as prepared, for other 12 hours. The product was separated by imposition of an external magnet and washed several times with water. The isolated material was then transformed into a red brown powder (final synthesis product) by drying and curing at 400 °C for 2 hours. The resulting nanopowder showed a magnetic response upon exposure to a magnetic field. The final mass of product was 2.0 g (12.5 mmol) of Fe_2_O_3_ and a yield of 68% was calculated. The nanoparticulated resulting material was characterized by zero field and in field (5 T) Mossbauer spectroscopy, FTIR spectroscopy, high resolution transmission electron microscopy, XRPD, magnetization measurements^102,103^ and the resulting material consisted of stoichiometric maghemite (γ-Fe_2_O_3_) with a mean diameter of 11 ± 2 nm, which is able to lead to the formation, upon sonication in water (Bransonic, mod. 221, 48 kHz, 50 W) of a stable colloidal suspension, without any organic or inorganic coating. The surface of these bare maghemite nanoparticles shows peculiar binding properties and can be reversibly derivatized with selected organic molecules. We called these naked nanoparticles Surface Active Maghemite Nanoparticles (SAMNs).

### Stability of SAMN suspensions

The stability of SAMN suspensions was evaluated in two different media suitable for *D*. *magna* culture: Aachener Daphnien Medium (ADaM) (pH 8.0, dry residue 587 mg L^−1^ ^51^) and oligomineral water Rocchetta (pH 7.9, dry residue 177 mg L^−1^). Dissolved oxygen was 7.3–7.8 mg L^−1^ in both media, where suspensions of seven scaled concentrations of SAMNs (10, 25, 50, 75, 100, 150 and 200 mg L^−1^) were prepared. The concentration of each suspension was checked immediately after preparation and after 1, 2, 3, 24 and 48 hours using a spectrophotometer (Cary 60, Agilent Technologies) set at 400 nm, where maximum absorbance of SAMNs in water lays^104^. SAMN dispersions were characterized by dynamic light scattering (DLS) at the temperature of 25 °C, with a Zetasizer Nano-S (Malvern) equipped with a HeNe laser (633 nm).

### Culture conditions

*Daphnia magna* clones were maintained in oligomineral water (Rocchetta) at 20 ± 1 °C, with a 16 h light (2.6 µEm^−2^ s^−1^): 8 h dark photoperiod. They were fed three times per week with *Scenedesmus dimorphus* (8 × 10^5^ cells mL^−1^). The alga was cultured in 2 L Bold Basal Medium (BBM)^105^ enriched with a vitaminic complex and 3 g of sterilized poultry dung, and suspended by bubbling filtered air at 24 °C, under continuous illumination (90 µEm^−2^ s^−1^). Before use for feeding *Daphnia* cultures, the chlorophyte was filtered through a 50 µm laboratory test sieve (Endecotts LTD, London, England), centrifuged at 3000 *g* for 10 min, suspended in 25% BBM at a concentration of 2 × 10^8^ cells mL^−1^ and stored at 4 ± 1 °C.

The algae used for the toxicity tests were from axenic cultures of *Pseudokirchneriella subcapitata* (strain UTEX 1648) in mid exponential growth phase. The cultures were maintained in 2 L BBM enriched with a vitaminic complex and suspended by bubbling filtered air at 24 °C, under continuous illumination (90 µEm^−2^ s^−1^).

The duckweed *Lemna minor* was axenically cultured in 100 mL of BBM at 24 °C and at a light intensity of 108 µEm^−2^ s^−1^. From a single isolated plant, randomly selected from the culturing flask, a stock culture was grown for three weeks before the initiation of the toxicity tests.

### Acute toxicity tests of SAMNs on *D*. *magna*

Acute toxicity tests were performed in accordance to the Guideline n. 202 “*Daphnia* sp., Acute Immobilization Test”^106^. The Rocchetta medium was used for controls and compared with SAMN dilutions on 4 groups of 5 young daphnids. In a preliminary test, performed without agitation, SAMN concentrations ranged between 2.5 and 40 mg L^−1^. Based on these preliminary results, further tests were run under agitation, and using the following SAMN concentrations: 1.25, 2.5, 5, 10 and 20 mg L^−1^. Daphnids were fed for about 1 h with dried Spirulina powder (15 mg in 100 mL Rocchetta) just before the starting of each experiment, and then each group (4 replicates, n = 5) was incubated in a beaker containing 50 mL of the test solution under the same conditions (light, temperature) used for culturing. Agitation was provided by gentle shacking at 100 rpm. Shaking, which is not envisaged by the OECD protocol, was chosen to minimize particle sedimentation and was considered tolerable as it reproduces random flows experienced by the animals in nature. Number of immobile daphnids was recorded after 24 and 48 h, the latter being the endpoint.

### Evaluation of the distribution of SAMNs in *D*. *magna*

Samples for electron microscopy analysis of cross sections of *D*. *magna* were prepared as follows: the specimens were fixed with 2.5% glutaraldehyde in 0.1 M sodium cacodylate buffer pH 7.4 for 1 hour at 4 °C, and post-fixed with a mixture of 1% osmium tetroxide. After three water washes, samples were dehydrated in a graded ethanol series and embedded in epoxy resin (Sigma-Aldrich). Semi-thin sections (1 µm) were obtained by an Ultrotome V (LKB) ultramicrotome, and counterstained with toluidine blue. *D*. *magna* specimens were observed by an XL 30 ESEM TMP microscope (Philips Electron Optics, Eindhoven, The Netherlands), equipped with an integrated energy dispersive X-ray spectrometer (XRF-EDS, EDAX system, Mahwah, NJ, USA). Moreover, *D*. *magna* cross sections (transversal and longitudinal) were analyzed by optical microscopy using a Leica DMR light microscope. Furthermore, living *D*. *magna* were filmed using a Leica MZ16 stereomicroscope equipped with a Leica DFC-480 digital camera.

### Evaluation of SAMN loading and depletion in *D*. *magna*

Adult daphnids (n = 190), two weeks of age, were collected and randomly allocated, in groups of 5, to 38 capped tubes containing 50 mL of a SAMN suspension (1 mg L^−1^) in Rocchetta medium. The tubes were shaken at 100 rpm and maintained under the same conditions (light, temperature) used for culturing. At 1 h intervals during the first 8 h incubation, and once again after a further 16 h incubation, two groups of daphnids were collected, weighed and frozen, for pending analyses. The remaining daphnids (n = 100) were transferred in a tank containing clean Rocchetta medium, fed with *S*. *dimorphus* (8 × 10^5^ cells mL^−1^) and kept under normal culture conditions for further 8 h during which, at 1 h intervals, 10 individuals were collected weighed and frozen, for pending analyses. The last 20 daphnids were not sampled but were checked for their health status for a further 12 days.

Total iron content in each sample was determined by Inductively Coupled Plasma Atomic-Emission Spectrometry (ICP-AES, Arcos by Spectro, Berwyn, PA, USA), after mineralization of the biological material with nitric acid.

### Measurement of the swimming activity of *D. magna* after exposure to SAMNs

Four groups of 5 daphnids (6 day of age) were allocated in Roux flasks (75 cm^2^), under the conditions previously described, and exposed for 48 h in the presence of 1.25 or 0.625 mg L^−1^ SAMNs, or used as controls (clean Rocchetta medium). The flasks were constantly shaken at 100 rpm throughout the test (see above). At the end of the test, each daphnid was individually transferred to a new flask containing clean Rocchetta medium and frontally filmed for 5 minutes. The central 3 min portion of the video sequence was analysed using Tracker software (freeware, opensourcephysics.org), which calculates the speed of the organism. Then, the data capturing the *x* and *y* positions provided by the software were exported to Microsoft Excel in order to measure the travelled distance.

### Effects of SAMNs on *D. magna* embryos

Gravid daphnids were collected from cultures and examined microscopically for the level of development of embryos in the brood chamber. About fifty specimens, carrying embryos in the early development (stage 1)^107^, were selected for the experiment. Embryos were extracted by immobilizing the head of the adult with a dissecting probe, while a second probe was used to gently separate the embryos^107^. Tests were performed in 24-well Suspension Culture Plate (Cellstar, Greinerbio-one) and 24 embryos were individually exposed in 1 mL to 5 SAMN suspensions, in the 1.25–20.0 mg L^−1^ concentration range, or to pure Rocchetta medium for control. Incubations lasted 3 days under the conditions previously described. After incubation, the number and the individual features of living and dead embryos were observed.

### Chronic toxicity test on *D. magna*

A chronic (21 days) semi-static test was performed in general accordance with the OECD Guideline n. 211 on ‘*D*. *magna* reproduction test’^85^. Based on the results from the acute toxicity test, five SAMN concentrations were assayed in the 0.078–1.25 mg L^−1^ range. For each SAMN concentration, 10 young daphnids (<24 h old) were individually allocated to beakers containing 50 mL solution, and incubated under the conditions already reported (see above). Solutions were renewed every other day by moving the daphnids to a new solution, and feed (*S*. *dimorphus*, 8 × 10^5^ cells mL^−1^) was supplied. Neonates were removed and counted. Incubation solutions were monitored for dissolved oxygen, using an YSI 85 Multiparameter Instrument (YSI Incorporated, Yellow Springs, OH, USA), and pH.

Sublethal effects on daphnid growth were determined as follows: sixty randomly selected juveniles (<24 h old), not intended for the test, were transferred in clean Rocchetta medium and fixed by progressive ethanol additions up to 70% concentration. Daphnid length was measured (from the top of the eye to the base of the tail spine) by a DMD108 Digital Microimaging Device (Leica Microsystems, Milano, Italy), and the average daily growth rate was calculated over the 21 d. period.

### Toxicity test of SAMNs on *Pseudokirchneriella subcapitata*

The toxicity of SAMNs on the freshwater green alga *Pseudokirchneriella subcapitata* was evaluated according to the EPA 797.1050-Algal acute toxicity test^108^. Algal inocula, corresponding to 10,000 cells mL^−1^, were grown in 125 mL Erlenmeyer flasks, containing 50 mL of BBM in the absence (controls) or in the presence of SAMNs. Five scaled concentrations, in the 1.25–20 mg L^−1^ SAMN concentration range, were tested. The test was carried out in triplicate, in axenic conditions. The flasks were incubated on a shaking (100 rpm) apparatus under the same conditions (light, temperature) used for culturing. After 24, 48, 72 and 96 hrs, the algal growth was measured by counting the cells in a Burker chamber.

### Toxicity test of SAMNs on *Lemna minor*

The growth inhibition test on *L*. *minor* was performed in accordance with the OECD guideline n. 221^109^. Roux flasks (75 cm^2^), equipped with vented caps, were filled with 100 mL of BBM in the absence (controls) or in the presence of SAMNs. Five scaled SAMN concentrations, in the 1.25–20 mg L^−1^ range, were tested in triplicate. The test started by introducing 9 *L*. *minor* fronds into each flask. Only plants with two or three fronds were chosen. The flasks were incubated under the same conditions used for culturing. After the exposure period (one week), the colonies were photographed using a Nikon Coolpix S3000 camera. The frond number was counted and the total frond area was measured using ‘ImageJ’ free software. Fronds from each flask were then blot dried on tissue paper and weighed (fresh weight).

### Data analysis

For the acute toxicity test on *D*. *magna*, EC_50_ and the associated 95% confidence interval were calculated using the trimmed Spearman-Karber Method^110^. Data on *D*. *magna* swimming activity, reproduction and growth, as well as data on *P*. *subcapitata* and *L*. *minor* growth, were analyzed using ANOVA to account for differences between groups, and expressed as means ± standard deviation.

## Electronic supplementary material

Supporting information

Supporting movie
