# Supplementary material for: The surface reactivity of iron oxide nanoparticles as a potential hazard for aquatic environments: A study on Daphnia magna adults and embryos
Source: Sci Rep. 2018 Aug 29;8:13017. doi: 10.1038/s41598-018-31483-6 (PMC6115473; doi:10.1038/s41598-018-31483-6)

Supplementary Information

The surface reactivity of iron oxide nanoparticles as a potential hazard for aquatic environments: A study on Daphnia magna adults and embryos

Massimiliano Magro^a,b^, Marco De Liguoro^a^, Eleonora Franzago^a^, Davide Baratella^a^, and Fabio Vianello^a,b,^*

*^a^ Department of Comparative Biomedicine and Food Science, University of Padua, Agripolis, Viale dell’Università 16, 35020 Legnaro, Italy*

*^b^ Regional Centre of Advanced Technologies and Materials, Department of Physical Chemistry and Experimental Physics, Faculty of Science, Palacky University, 17 Listopadu 1192/12, 771 46 Olomouc, Czech Republic*

***** **Correspondence and requests for materials should be addressed to F.V. (**Department of Comparative Biomedicine and Food Science, University of Padua, Agripolis, Viale dell'Università 16, 35020 Legnaro, Italy, ph: 0039-049-8272638, e-mail: fabio.vianello@unipd.it)

**Supplementary Figure S1.** Swimming trajectory in vectors (three minutes path) of a *D. magna* individual. a) control; b) after 48 h exposure to 1.25 mg L^-1^ SAMNs. Image generated by Tracker® software.


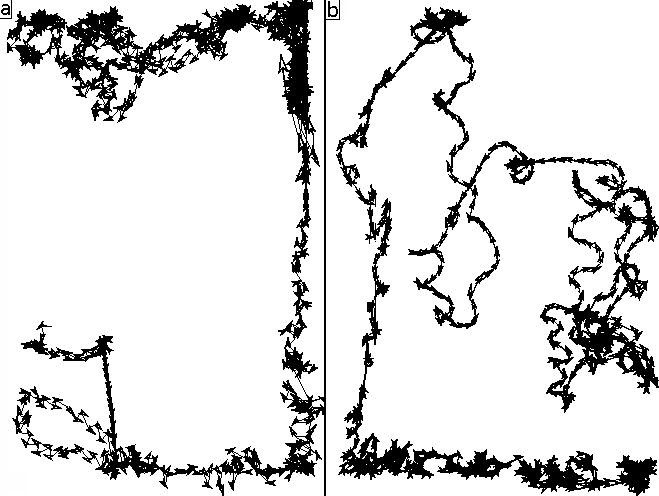


**Supplementary Figure S2.** Amplitudes of swimming speed of *D. magna* individuals exposed to SAMNs. (a) animals (n = 20) exposed to 1.25 mg L^-1^ SAMNs; (b) controls. Single tracks were superimposed. Graphs were generated by Tracker® software.


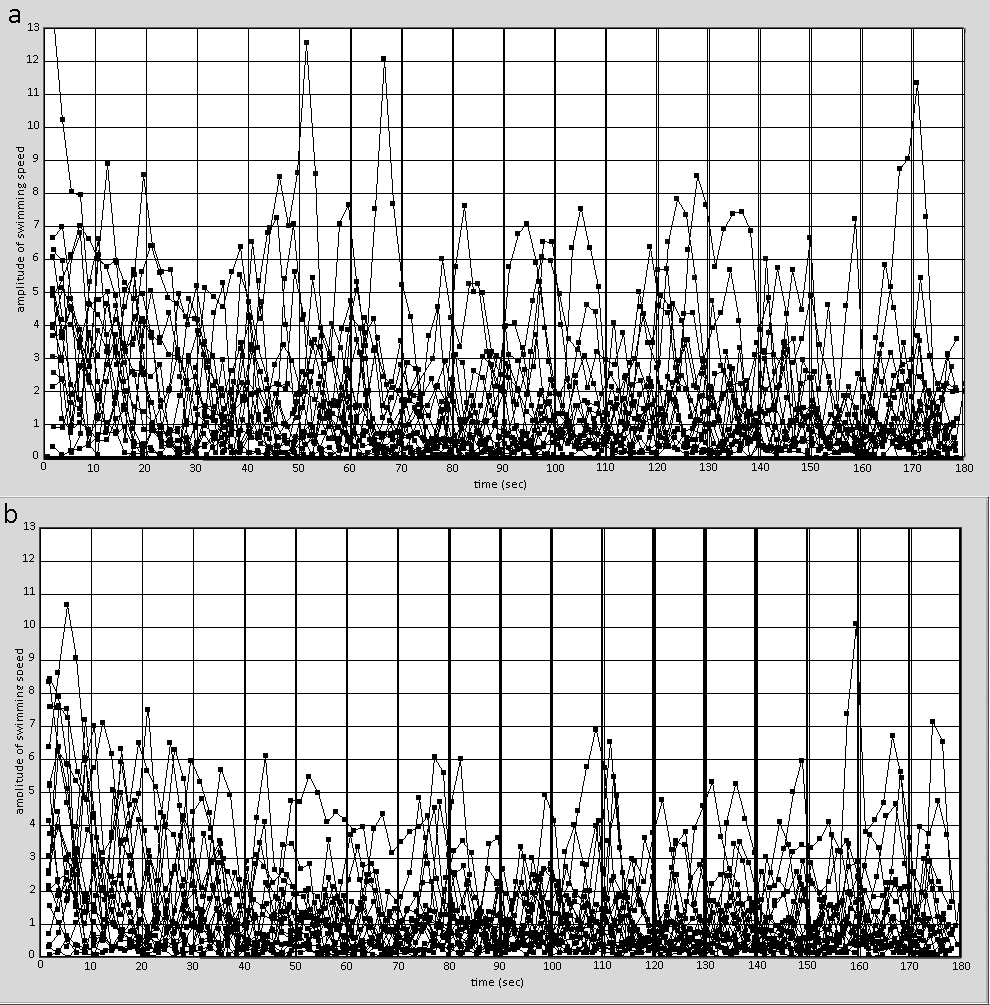

Supplement: Supplementary file 1 — Supporting information [file 41598_2018_31483_MOESM1_ESM.docx]
